# Dexmedetomidine ameliorates x-ray-induced myocardial injury via alleviating cardiomyocyte apoptosis and autophagy

**DOI:** 10.1186/s12872-024-03988-8

**Published:** 2024-06-26

**Authors:** Runze Zhang, Kangjie Xie, Yanhong Lian, Shufang Hong, Yuntian Zhu

**Affiliations:** Department of Anesthesiology, Zhejiang Cancer Hospital, No. 1 East Banshan Road, Gongshu District, Hangzhou, Zhejiang 310022 China

**Keywords:** X-ray-induced myocardial injury, Dexmedetomidine, Apoptosis, Autophagy

## Abstract

**Background:**

Radiotherapy is a primary local treatment for tumors, yet it may lead to complications such as radiation-induced heart disease (RIHD). Currently, there is no standardized approach for preventing RIHD. Dexmedetomidine (Dex) is reported to have cardio-protection effects, while its role in radiation-induced myocardial injury is unknown. In the current study, we aimed to evaluate the radioprotective effect of dexmedetomidine in X-ray radiation-treated mice.

**Methods:**

18 male mice were randomized into 3 groups: control, 16 Gy, and 16 Gy + Dex. The 16 Gy group received a single dose of 16 Gy X-ray radiation. The 16 Gy + Dex group was pretreated with dexmedetomidine (30 µg/kg, intraperitoneal injection) 30 min before X-ray radiation. The control group was treated with saline and did not receive X-ray radiation. Myocardial tissues were collected 16 weeks after X-ray radiation. Hematoxylin-eosin staining was performed for histopathological examination. Terminal deoxynucleotidyl transferase dUTP nick-end labeling staining was performed to assess the state of apoptotic cells. Immunohistochemistry staining was performed to examine the expression of CD34 molecule and von Willebrand factor. Besides, western blot assay was employed for the detection of apoptosis-related proteins (BCL2 apoptosis regulator and BCL2-associated X) as well as autophagy-related proteins (microtubule-associated protein 1 light chain 3, beclin 1, and sequestosome 1).

**Results:**

The findings demonstrated that 16 Gy X-ray radiation resulted in significant changes in myocardial tissues, increased myocardial apoptosis, and activated autophagy. Pretreatment with dexmedetomidine significantly protects mice against 16 Gy X-ray radiation-induced myocardial injury by inhibiting apoptosis and autophagy.

**Conclusion:**

In summary, our study confirmed the radioprotective effect of dexmedetomidine in mitigating cardiomyocyte apoptosis and autophagy induced by 16 Gy X-ray radiation.

## Background

Radiotherapy serves as a crucial adjuvant therapy for patients with cancer, with approximately two-thirds of patients with solid tumors receiving radiotherapy as part of their treatment [1]. However, in addition to targeting tumor cells, radiotherapy has the potential to cause damage to normal tissues or organs [2]. Thoracic radiotherapy-induced damage to myocardium tissue and heart valves is called radiation-induced heart disease (RIDH) [3, 4], with the first documented case of radiotherapy-induced cardiac mortality dating back to 1963 [5]. Recent clinical studies have demonstrated an elevated risk of heart disease-related deaths following radiation therapy [6]. However, up to now, there are still no clear treatment options to prevent or avoid the development of RIHD [7]. Therefore, the prevention and management of RIHD have become urgent clinical problems.

Oxidative stress is the main mechanism in RIHD [4]. Previous studies have demonstrated that reactive oxygen species generated after radiation lead to inflammatory responses, apoptosis, and autophagy [8–10]. Among these processes, autophagy serves as an internal waste reduction and recycling system within cells, playing a crucial role in maintaining cell homeostasis and adapting to physiological stress [11]. Due to the different cell types and environments, autophagy shows different functions: protective autophagy and autophagic cell death. On the one hand, autophagy protects tumor cells against radiation-induced cell injury, on the other hand, autophagy induces cell death to improve the radiosensitivity of tumor cells [12]. In myocardial injury, activated autophagy plays a critical role by promoting myocardial injury during ischemia/reperfusion (I/R)-induced myocardial injury [13]. In sepsis-induced myocardial injury, activated autophagy can lead to the induction of inflammation and tissue apoptosis [14]. However, the role of autophagy in radiation-induced myocardial injury has not been reported yet.

Dexmedetomidine (Dex), a highly selective alpha 2-adrenergic receptor agonist, is a widely used agent in clinical anesthesia [15]. Increasing evidence suggests that Dex has protective effects on organ damage caused by radiotherapy. For example, Dex decreases tubular epithelial apoptosis to show a protective effect in X-ray-induced testicular damage [16]. In X-ray radiation-induced parotid damage, Dex acts as a promising antioxidant agent [17]. In addition, the protective effects of Dex are also seen in myocardial injury [18–20]. Dex has also been found to be a regulator of autophagy. Through inhibiting autophagy, Dex showed neuroprotective effects in neurological injuries [21]. While Yu et al. found that Dex enhanced autophagy to alleviate the inflammatory responses in liver injury [22]. Therefore, we hypothesized that Dex may play a protective role in radiation-induced myocardial injury, and whether this protective effect is associated with Dex-mediated autophagy needs more studies.

In order to verify our hypothesis, we established the X-ray radiation-induced myocardial injury model in this study. Through in vivo and in vitro experiments, we aimed to confirm whether Dex plays a role in alleviating X-ray radiation-induced myocardial injury by regulating autophagy.

## Methods

### Mice and experimental protocol

Eighteen male C57BL/6 mice (8–10 weeks old, 20 ± 2 g) were purchased from Shanghai Model Organisms Center, Inc. (Shanghai, China), allowed to access food and water ad libitum and maintained under a 12 h dark/light cycle at 22 °C to 25 °C. These mice were randomly divided into 3 groups (*n* = 6 for each group): control, 16 Gy, and 16 Gy + Dex. Mice in the control group did not receive X-ray radiation or intraperitoneal injection of Dex. Mice in the 16 Gy group were exposed to 6 MV X-ray beam energy with 16 Gy dose (radiation area: the chest includes the heart region; radiation field: 10.6 mm × 15 mm) once on the first day as previously described [23]. Mice in 16 Gy + Dex were given Dex (30 µg/kg, intraperitoneal injection) 30 min before the 16 Gy X-ray radiation (the dose of Dex is referred from previous studies [24, 25]). Besides, mice in the control and 16 Gy group were given saline in a volume equivalent to that given to mice in the 16 Gy + Dex group.

Sixteen weeks later, the mice were anesthetized and sacrificed by cervical dislocation. Their hearts were isolated and fixed in a 10% formaldehyde solution for 24 h. Subsequently, the samples were sectioned to obtain 1.5 mm thick myocardial tissue using a rotary microtome (RM 2016, Leica Biosystems, Germany). After being fixed in 10% formaldehyde solution for 12 h, the sections were embedded in paraffin for hematoxylin-eosin (HE) staining, terminal deoxynucleotidyl transferase dUTP nick-end labeling (TUNEL) staining, immunohistochemistry (IHC) staining, and western blot analysis. All procedures in this study were approved by the Ethics Committee of Zhejiang Cancer Hospital and conducted according to standard institutional guidelines.

### HE staining

For histopathological examination, HE staining was used. Briefly, 5 μm thick sections of paraffin-embedded heart tissues were prepared and stained with Mayer’s Hematoxylin (Sigma, St. Louis, USA) for 5 min followed by counterstaining with Eosin for 5 min (Sigma). Subsequently, the sections were dehydrated, sealed, and observed under a microscope (BX53, Olympus, Tokyo, Japan).

### IHC staining

For immunohistochemistry, 4 μm sections of paraffin-embedded heart tissues were deparaffinized, rehydrated, and subjected to antigen retrieval. Subsequently, the sections were incubated with bovine serum albumin, followed by overnight incubation at 4 °C with antibodies against CD34 molecule (CD34, 1:100, ab81289, Abcam, Cambridge, USA) and von Willebrand factor (vWF, 1: 100, ab287962, Abcam). After further washing steps, the sections underwent incubation with a secondary antibody (Abcam) and diaminobenzidine reagent (DAKO, Glostrup, Denmark), followed by counterstaining with hematoxylin. Finally, the sections were observed under a microscope (BX53, Olympus). Images were randomly obtained from five fields for each sample. The quantitative analysis was performed by calculating the ratios of integrated optical density to the positive area in Image J2 software (Version 2.14.0/1.54f).

### TUNEL staining

To identify myocardial apoptosis, a TUNEL assay was performed. The apoptotic cardiomyocytes were labeled using the colorimetric TUNEL system (Promega, Madison, USA) according to the manufacturer’s protocol. The positively stained cells were counted in five randomly selected fields in a blinded manner under microscope.

### Cell cultures and treatment

Mouse cardiomyocyte HL-1 was obtained from Procell (Wuhan, China) and maintained in minimum essential medium (MEM, containing non-essential amino acids, Procell) supplemented with 10% fetal bovine serum (FBS, Procell) and 1% penicillin-streptomycin (Procell) at 37 °C with 5% CO_2_.

To simulate X-ray radiation-induced myocardial injury, HL-1 cells were exposed to a single dose of 16 Gy X-ray. To verify the radioprotective of Dex in vitro, 5µM Dex was added to the culture medium before radiation. After 48 h, the cells were collected for subsequent experiments.

### Cell viability assay

The cell viability was analyzed using a cell counting kit-8 (CCK-8) assay kit (MedChemExpress, Monmouth Junction, USA). Briefly, cells were seeded in a 96-well plate (10, 000 cells/well) and subjected to different treatments. After 48 h, 10µL CCK-8 solution was added to each well. Following a 2-hour incubation, cell absorbance was detected at 450 nm with a microplate reader (Multiskan MK3, Thermo Fisher Scientific, Waltham, USA).

### Flow cytometry

The cell apoptosis was analyzed using an Annexin V-fluorescein isothiocyanate (FITC) apoptosis detection kit (Beyotime, Shanghai, China). Briefly, cells were incubated with Annexin V-FITC binding buffer and propidium iodide solution. After 20 min, cell apoptosis was detected using flow cytometry (CytoFLEX, Beckman Coulter, Miami, USA).

### Western blot analysis

The proteins were extracted from tissues and cells using RIPA lysis buffer (Beyotime, Shanghai, China). Then, equal amounts of proteins were separated by sodium dodecyl sulfate-polyacrylamide gel electrophoresis, transferred onto polyvinylidene fluoride membranes, and separately incubated with the following primary antibodies: BCL2 apoptosis regulator (Bcl-2, AF6139, 1:1000, Affinity, USA), BCL2-associated X (Bax, AF0120, 1:1000, Affinity), microtubule-associated protein 1 light chain 3 (LC3, AF5402, 1:1000, Affinity), beclin 1 (Beclin-1, AF5128, 1:1000, Affinity), sequestosome 1 (p62, AF5384, 1:1000, Affinity), and glyceraldehyde-3-phosphate dehydrogenase (GAPDH, ab9485, 1:1000, Abcam). The membranes were washed and incubated with the corresponding secondary antibody (goat anti-rabbit IgG horseradish peroxidase, S0001, 1:5000, Affinity) for 2 h at 37 °C. Finally, proteins were visualized using an enhanced chemiluminescence reagent (Millipore, USA). Relative protein levels were quantified by densitometry using Image J2 software.

### Statistical analysis

Each experiment was repeated at least three times and data were expressed as mean ± standard deviation. Statistical analysis was performed in GraphPad software (version 9.5). The post hoc power was calculated with the G*Power (version 3.1.9.2). Normality was assessed using Shapiro-Wilk tests and equal variances were assessed using the Brown-Forsythe test. Differences among the three groups were analyzed using one-way analysis of variance with Tukey’s post hoc test. The significance level was set at 0.05.

## Results

### Effects of dexmedetomidine on myocardial pathological injuries of x-ray-induced myocardial injury mice

**Fig. 1:**
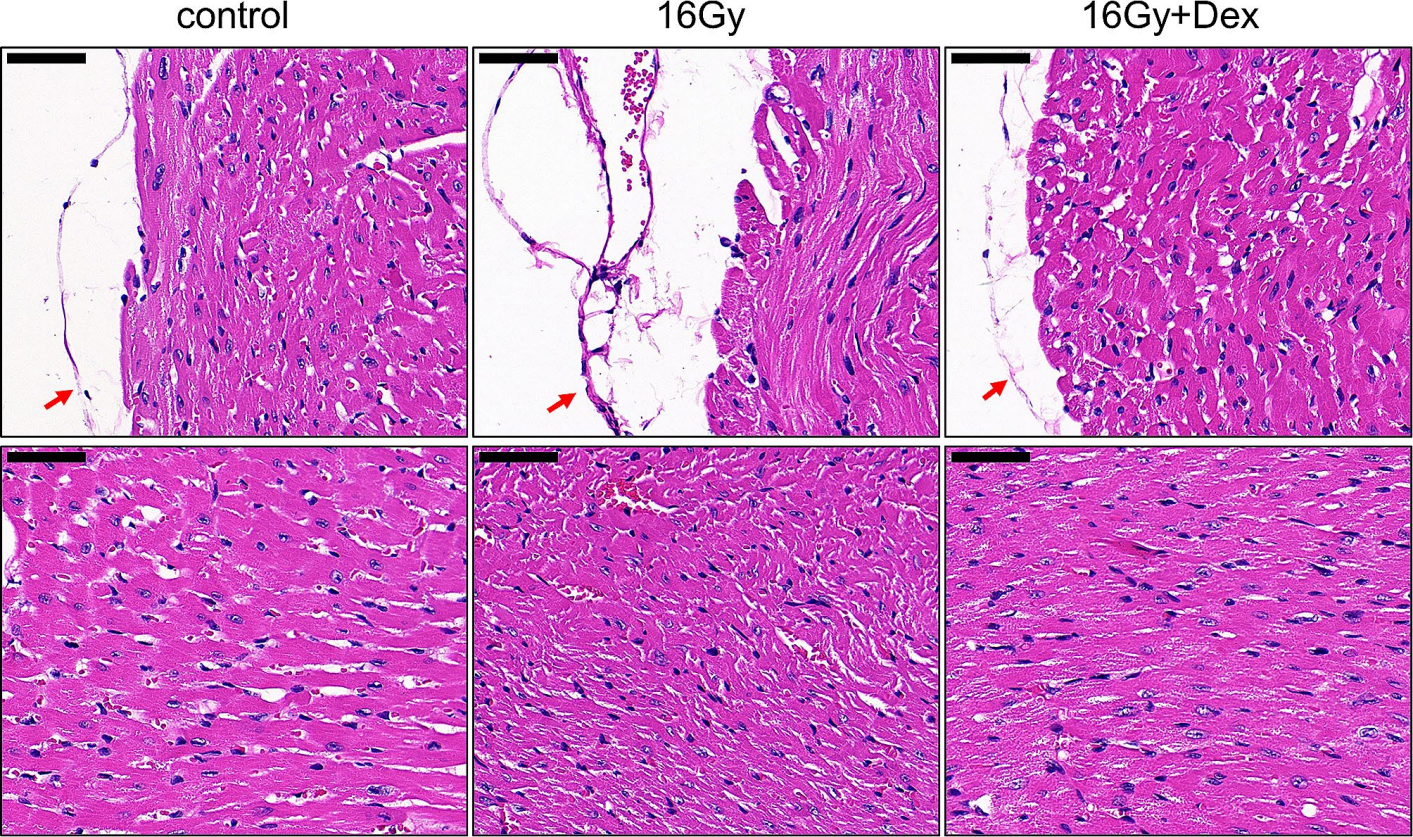
Representative images of Hematoxylin-Eosin staining results. HE staining of the epicardium and myocardial tissues of mice at 16 weeks after radiation. Upper: epicardial thickness in each group (red arrows); Lower: histological changes in each group. After 16 Gy X-ray radiation, the epicardial thickness increased and the myocardial tissue showed extensive fibrous thickening. Dex pretreatment restored epicardial thickness and myocardium structure. *n* = 6 for each group. Dex: dexmedetomidine. Scale bar: 50 μm

We first observe the histopathological changes by HE staining. As shown in Fig. 1, in the control group, there was no exudation or hyperemia edema in the myocardial tissues. The muscle fibers displayed a normal arrangement and a distinct structure. In the 16 Gy group, the myocardial tissue demonstrated widened myocardial space, disordered muscle fiber arrangement, indistinct transverse stripes, and fibrin exudation. The morphology of myocardial tissues in 16 Gy + Dex group was close to that of the control group, suggesting that Dex pretreatment could alleviate the tissue deterioration. We also noted that 16 Gy X-ray radiation caused the thickening of the epicardium, which was mitigated by Dex pretreatment (arrows in Fig. 1). These findings suggested that Dex could alleviate the 16 Gy X-ray radiation-induced myocardial injury in mice.

### Effects of dexmedetomidine on vascular endothelial cells injury of x-ray-induced myocardial injury mice

**Fig. 2:**
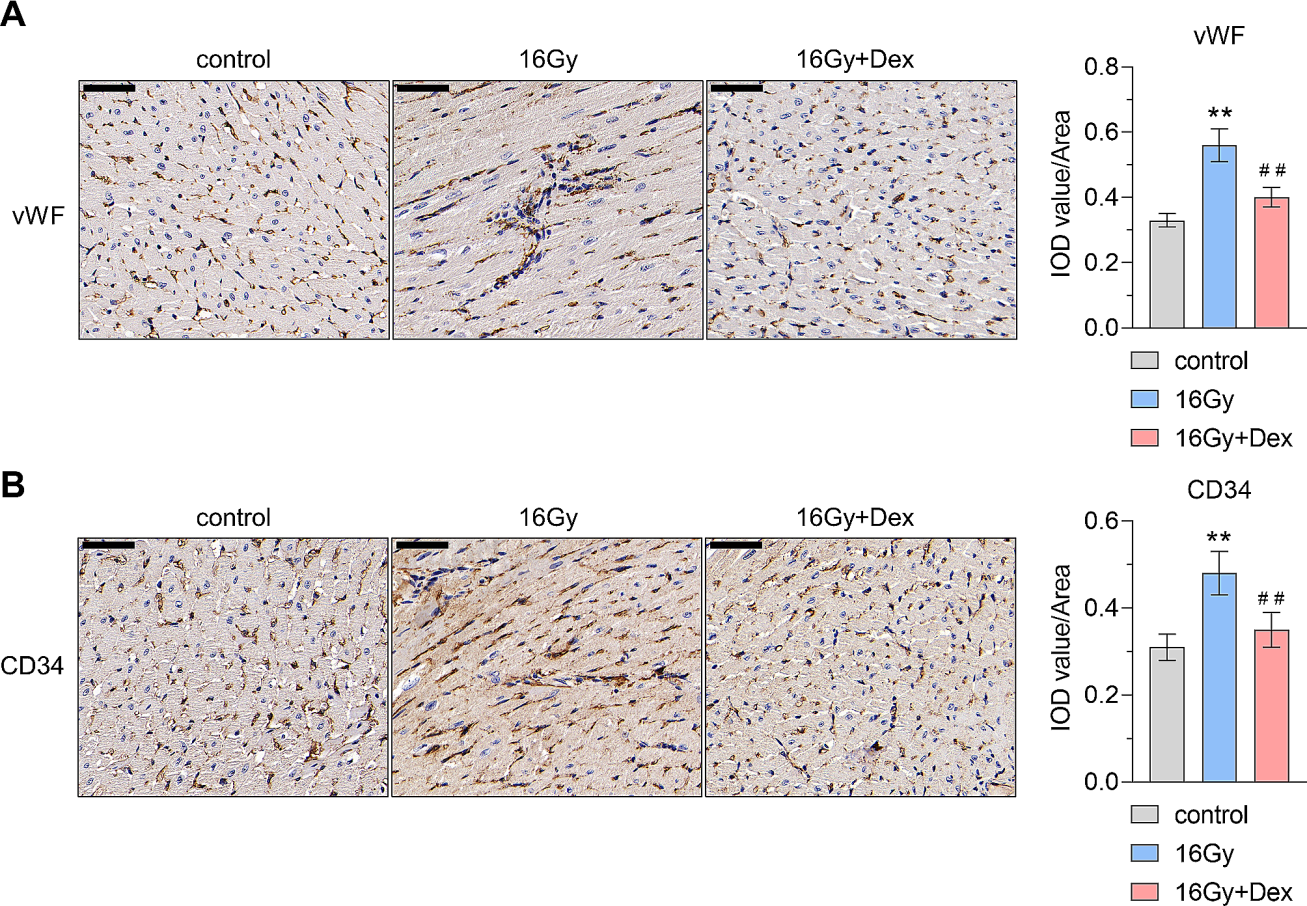
Representative images of immunofluorescence staining results. Immunohistochemistry staining for CD34 molecule (CD34) and von Willebrand factor (vWF) in myocardial tissues and quantification of CD34 **(A)** and vWF **(B)** expression levels in myocardial tissues. The expression of CD34 and vWF is increased when the microvascular structure is damaged, which can be used as biomarkers for endothelial cell injury. *n* = 6 for each group. Dex: dexmedetomidine; IOD: integrated optical density. Scale bar: 50 μm. Data were represented as mean ± standard deviation, ** *P* < 0.01 vs. control group, ## *P* < 0.01 vs. 16 Gy group. For CD34 quantification analysis, effect size = 4.23, power = 1; For vWF quantification analysis, effect size = 2.57, power = 1

To further investigate the microvasculature damage, the expression of CD34 and vWF was evaluated by IHC staining. CD34 and vWF are endothelial markers and could reflect endothelial cell damage [26–28]. Analysis of the IHC staining results showed higher expression of CD34 and vWF in the 16 Gy group compared to the control group, suggesting that 16 Gy X-ray radiation could induce vascular endothelial cell injury. However, Dex pretreatment significantly attenuated the 16 Gy X-ray radiation-induced upregulation of CD34 and vWF in myocardial tissues (Fig. 2A-B). These findings suggested that Dex exerts a protective effect on X-ray-induced vascular endothelial cell injury.

### Effects of dexmedetomidine on myocardial apoptosis of x-ray-induced myocardial injury mice

**Fig. 3:**
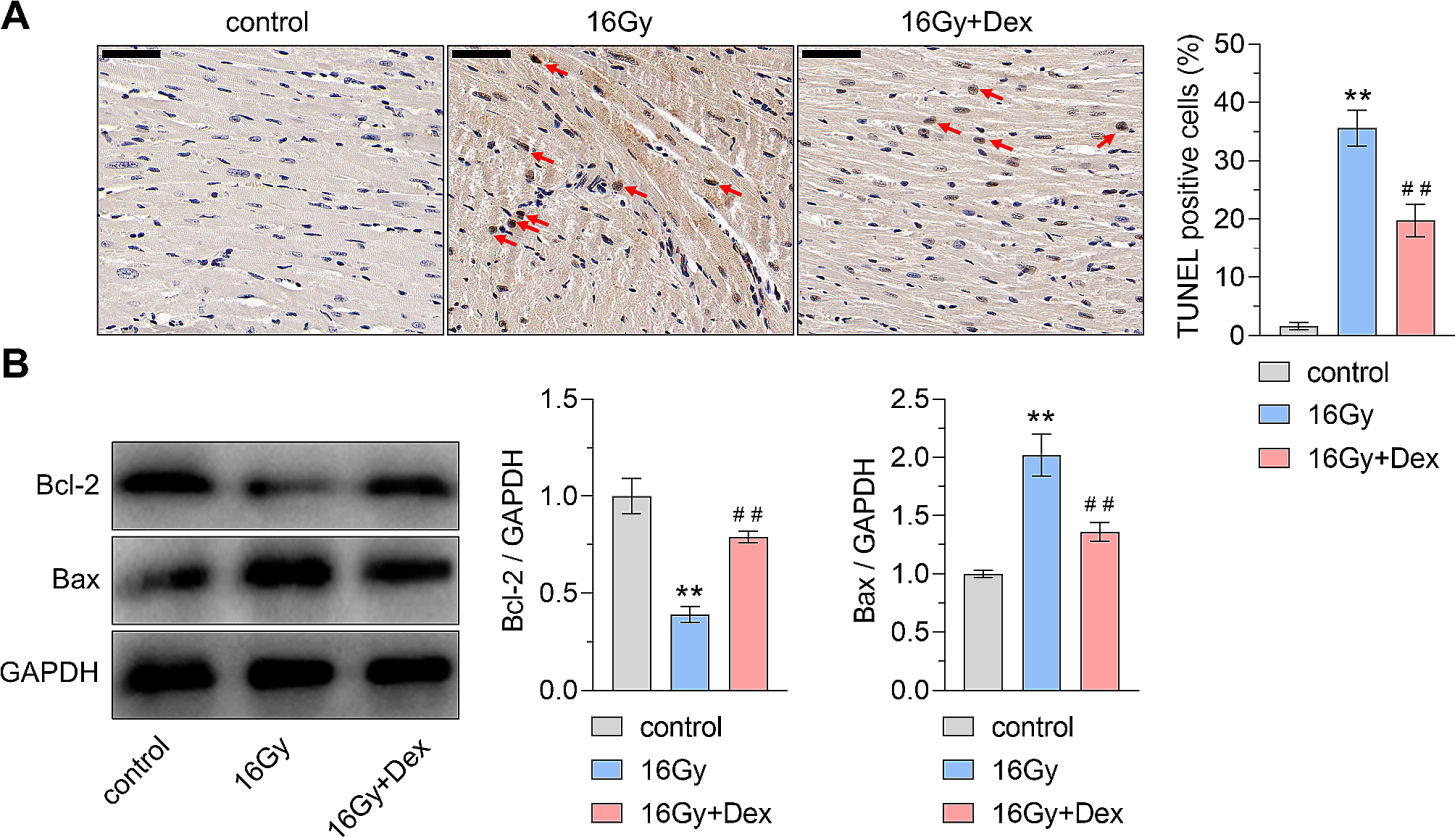
Effects of dexmedetomidine on regulating myocardial apoptosis. **(A)** Representative images and quantitative analysis of TUNEL staining results. The TUNEL-positive cells were shown by red arrows. ** *P* < 0.01 vs. control group, ## *P* < 0.01 vs. 16 Gy group; effect size = 10.81, power = 1.00. **(B)** Western blot analysis and densitometry quantification of the protein expression of BCL2 apoptosis regulator (Bcl-2) and BCL2-associated X (Bax). ** *P* < 0.01 vs. control group, ## *P* < 0.01 vs. 16 Gy group. For Bcl-2 quantification analysis, effect size = 2.57, power = 1; for Bax quantification analysis, effect size = 5.99, power = 1. *n* = 6 for each group. Dex: dexmedetomidine. Scale bar: 50 μm. Data were represented as mean ± standard deviation

Next, we detected the protective effect of Dex on myocardial apoptosis. TUNEL staining results showed an upregulation of TUNEL-positive cells in myocardial tissues following exposure to 16 Gy X-ray, indicating heightened myocardial apoptosis. However, mice pretreated with Dex in the 16 Gy + Dex group exhibited a reduction in TUNEL-positive cells compared to those in the 16 Gy group (Fig. 3A). Western blot results further confirmed the effects of Dex on cell apoptosis. The expression of pro-apoptosis protein Bax was upregulated by 16 Gy X-ray treatment, and Dex pretreatment suppressed the Bax expression. Oppositely, the expression of anti-apoptotic protein Bcl-2 was downregulated by 16 Gy X-ray treatment, and Dex pretreatment promoted the Bax expression (Fig. 3B). These results indicated that dexmedetomidine exerts radioprotective effects through attenuation of cell apoptosis.

### Effects of dexmedetomidine on autophagy in x-ray-induced myocardial injury mice

**Fig. 4:**
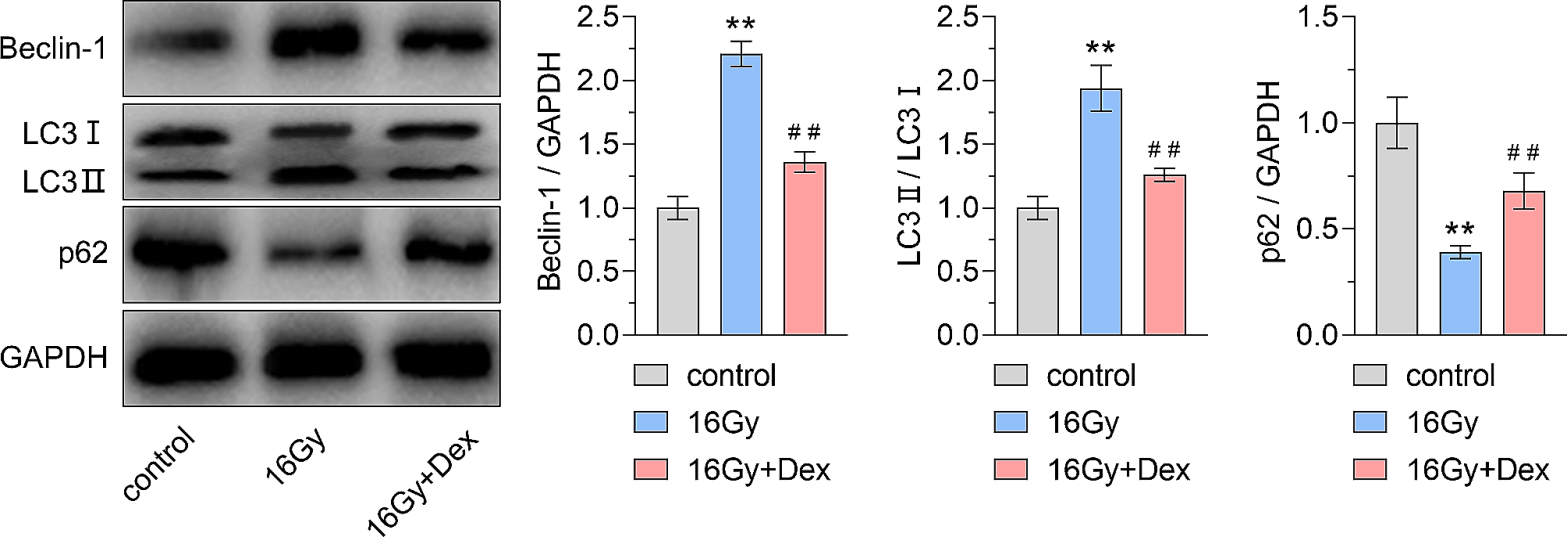
Western blot analysis and densitometry quantification of the protein expression of Beclin-1, LC3, and p62. Representative western blot and the quantitative analysis of the autophagy-related proteins beclin 1 (Beclin-1), microtubule-associated protein 1 light chain 3 (LC3), and sequestosome 1 (p62). ** *P* < 0.01 vs. control group, ## *P* < 0.01 vs. 16 Gy group. For Beclin-1 quantification analysis, effect size = 8.68, power = 1; for LC3 quantification analysis, effect size = 5.01, power = 1; for p62 quantification analysis, effect size = 5.35, power = 1. *n* = 6 for each group *n* = 6 for each group. Dex: dexmedetomidine. Data were represented as mean ± standard deviation

As previously reported, autophagy is crucially involved in the protective effect of Dex [29]. In this study, we detected the expression of autophagy-related proteins LC3, Beclin-1, and p62 through western blot assay. Compared with the control group, our results showed that exposure to 16 Gy X-ray radiation promoted the Beclin-1 expression and increased the LC3 II/I ratio, while concurrently downregulating p62 expression. Dex pretreatment inhibited the Beclin-1 expression and reduced the LC3 II/I ratio, while promoting the p62 expression (Fig. 4). These results indicated that the radioprotective effects of dexmedetomidine were partially due to autophagy inhibition.

### Effects of dexmedetomidine on cell viability, apoptosis, and autophagy in x-ray-treated cardiomyocyte

**Fig. 5:**
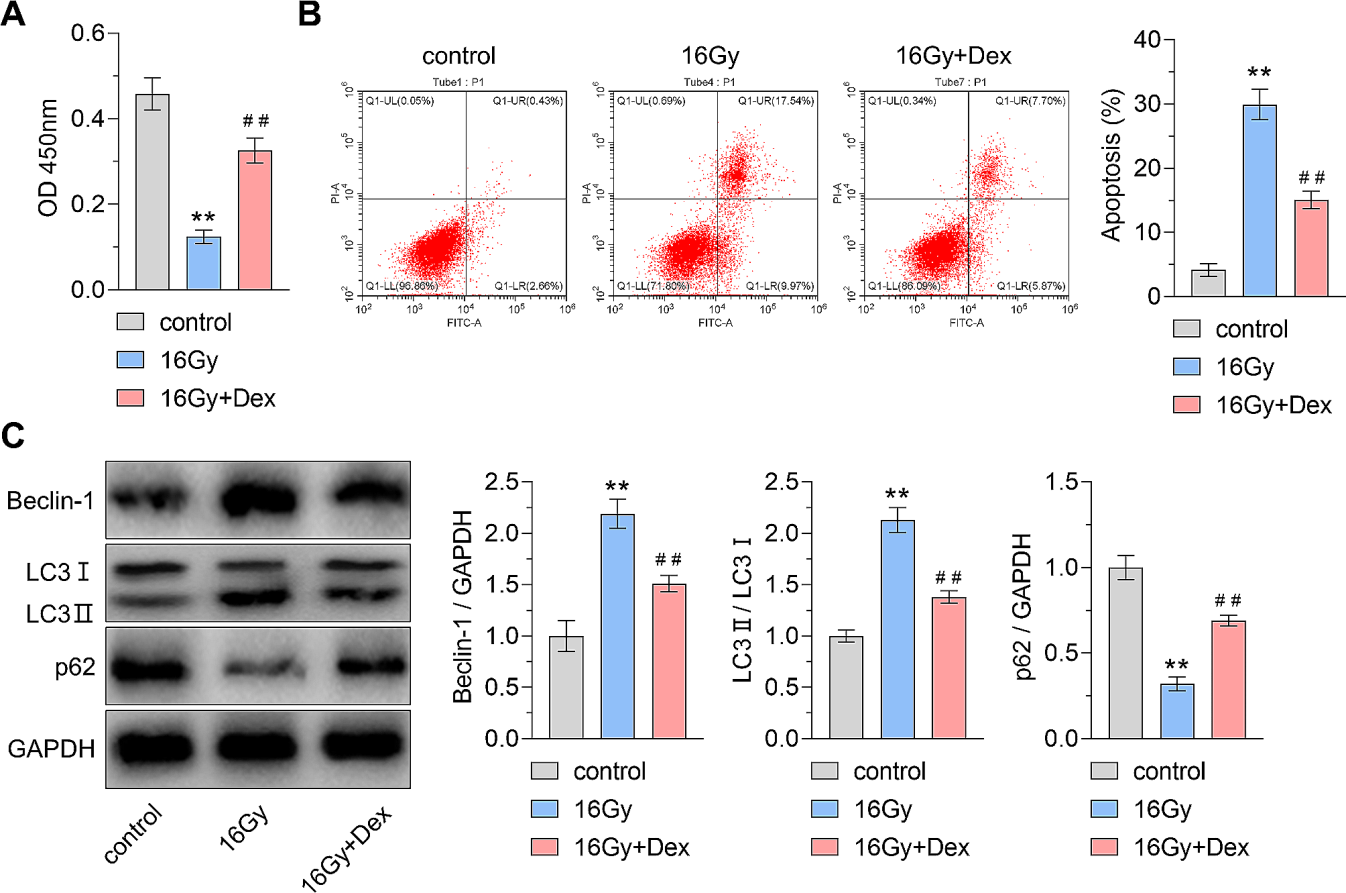
Effects of dexmedetomidine in X-ray-treated cardiomyocytes. **(A)** CCK-8 assay was applied to detect cell viability. ** *P* < 0.01 vs. control group, ## *P* < 0.01 vs. 16 Gy group; effect size = 8.03, power = 1 **(B)** Flow cytometry was used to detect cell apoptosis. ** *P* < 0.01 vs. control group, ## *P* < 0.01 vs. 16 Gy group; effect size = 10.87, power = 1 **(C)** Western blot analysis and densitometry quantification of the protein expression of beclin 1 (Beclin-1), microtubule-associated protein 1 light chain 3 (LC3), and sequestosome 1 (p62). ** *P* < 0.01 vs. control group, ## *P* < 0.01 vs. 16 Gy group. For Beclin-1 quantification analysis, effect size = 6.68, power = 1; for LC3 quantification analysis, effect size = 8.89, power = 1; for p62 quantification analysis, effect size = 9.97, power = 1. Dex: dexmedetomidine. Data were represented as mean ± standard deviation

We then verified the radioprotective effects of Dex in vitro. The cell viability was detected through a CCK-8 assay. As shown in Fig. 5A, cell viability was reduced in the 16 Gy group compared to the control group. However, pretreatment with Dex reversed the radiation-induced suppression of cell viability caused by 16 Gy X-ray. Conversely, treatment with 16 Gy X-rays led to an increase in the rate of cell apoptosis, which was mitigated by Dex pretreatment (Fig. 5B). Western blot analysis revealed upregulation of Beclin-1 expression and LC3 II/I ratio, as well as downregulation of p62 expression in the 16 Gy group compared to the control group. Notably, Dex pretreatment decreased Beclin-1 expression and LC3 II/I ratio, while promoting p62 expression (Fig. 5C). These findings confirmed that the radioprotective effects of dexmedetomidine were through regulating apoptosis and autophagy in vitro.

## Discussion

This study was conducted to investigate the role of Dex in X-ray radiation-induced myocardial injury. Through animal and cell experiments, we found that Dex pretreatment suppressed cardiomyocyte apoptosis and ameliorated the X-ray radiation-induced myocardial injury. Furthermore, we found that the radioprotective role of Dex was related to the inhibition of autophagy.

In recent years, radiotherapy has emerged as a crucial modality for cancer treatment. However, exposure of the chest to radiation can lead to damage in vascular endothelial cells and cardiomyocytes, and this damage is related to the radiation type and dose [30]. Clinical studies revealed that 1–4 Gy radiation promotes the development of cardiovascular diseases and inflammation, 4–8 Gy radiation increases the possibility of myocardial infarction, and > 8 Gy radiation causes myocardial fibrosis [31–34]. In an animal study, it was found that cardiac radiation exposure (10–20 Gy) resulted in cardiomyocyte hypertrophy, left ventricular diastolic dysfunction, and myocardial fibrosis [35]. Consistent with previous studies, our results showed that 16 Gy X-ray radiation resulted in the thickening of the epicardium and disorder of myocardial fiber arrangement. In addition, we performed the IHC staining to detect the CD34 and vWF expression in myocardial tissues. CD34 is an adhesion molecule belonging to the cadherin family. It can be selectively expressed on the surface of hematopoietic stem cells and acts as a major molecule involved in many pathophysiological processes such as intercellular signaling, immune response, inflammation, and coagulation [36]. vWF is produced and secreted by vascular megakaryocytes and endothelial cells and is involved in hemostasis [37]. In cases of endothelial cell injury, there is an elevation in plasma vWF levels [38]. Furthermore, with progressive vascular leakage leading to microvascular structural damage, the expressions of CD34 and vWF are upregulated as indicators of endothelial cell damage [26–28]. Herein, we found that both CD34 and vWF expression were upregulated by 16 Gy X-ray radiation, suggesting the occurrence of vascular endothelial cell injury.

The heart is an organ with high oxygen consumption, and cardiomyocytes contain a large number of mitochondria [35]. In the process of radiation-induced damage, mitochondria are particularly susceptible [39]. A previous study revealed that radiation-induced mitochondria damage could induce cell apoptosis, which is an important pathological mechanism in radiation-induced tissue damage [40, 41]. Here, through TUNEL staining, we confirmed that 16 Gy X-ray radiation facilitated cardiomyocyte apoptosis. Besides, mitochondrial damage is related to changes in levels of Bax and Bcl-2 [42]. Bax and Bcl-2 are two important genes in regulating apoptosis. The ratio between Bax/Bcl-2 proteins was a key factor in promoting apoptosis [43]. In this study, we found that 16 Gy X-ray radiation promoted Bax expression while inhibiting Bcl-2 expression, which was consistent with the previous research [44].

Besides promoting apoptosis, mitochondrial damage may also lead to autophagy activation [4]. The function of autophagy in myocardial injury has been controversial. Chen et al. found that autophagy is activated in myocardial I/R injury [45]. Xing et al. found that autophagy is blocked in myocardial I/R injury, and autophagy restoring rescues heart function [46]. The reason for this contradiction may be the two sides of autophagy. Under normal circumstances, autophagy can degrade damaged organelles and harmful proteins to recover nutrients and generate energy, thus promoting cell and tissue survival. However, with the increase of the stimulation intensity or time, the level of basal autophagy will gradually increase to form excessive autophagy, which leads to impaired cell function and autophagic death [47]. In this study, under 16 Gy X-ray radiation, Beclin-1 expression and LC3 II/I ratio increased, while the p62 expression decreased. These results suggested that autophagy was active in 16 Gy X-ray radiation-induced myocardial injury.

Dex is currently used for its excellent sedation and analgesia with minimal cardiovascular effects [48]. Previous research has clarified the important role of Dex in myocardial injury. Through reducing ferroptosis, Dex alleviates sepsis‑induced myocardial cellular injury [49]. Another study presented that Dex pretreatment attenuates myocardial I/R injury by relieving endoplasmic reticulum stress [50]. Wu et al. found that Dex protects against myocardial I/R injury via ameliorating oxidative stress and cell apoptosis [51]. However, no previous studies have examined the effectiveness of Dex in protecting radiation-induced myocardial injury, which this study demonstrates. By pretreatment with Dex, the myocardial injury and apoptosis induced by 16 Gy X-ray were attenuated. Furthermore, we found that the protective effect of Dex on X-ray radiation-induced myocardial injury was through inhibiting autophagy. As Dex has been extensively used in clinical practice [52], our data may extend the possibility of using Dex for radiation-induced myocardial injury.

Some limitations of the current study should be admitted. First, only 6 mice were used in each group because of insufficient expenditure. The small sample size reduced the statistical power of the study results. To overcome this limitation, the post hoc power of the results was calculated and was high. Second, we did not test other treatment doses or duration of Dex. Whether Dex pretreatment displays a dose- or time-dependent effect deserves further clarification. Third, our study did not include a molecular mechanism investigation. Li et al. found that dexmedetomidine activates the phosphatidylinositol-3-kinase/protein kinase B/mammalian target of rapamycin pathway to attenuate autophagy in cerebral ischemia-reperfusion injury [53]. Another study reported that dexmedetomidine decreases reactive oxygen species production and nuclear factor kappa-B proteins to inhibit the activation of autophagy in traumatic brain injury [54]. These results provide possible molecular mechanisms for the inhibition of autophagy by dexmedetomidine in radiation-induced myocardial injury, which will be investigated in further studies.

## Conclusion

Our study demonstrated that dexmedetomidine protected against 16 Gy X-ray radiation-induced myocardial injury by inhibiting apoptosis and autophagy. Our results may provide a new approach to the prevention of radiation-induced heart disease.

## Data Availability

The datasets used and analyzed during the current study are available from the corresponding author upon reasonable request.

## Abbreviations

RIHD: radiation-induced heart disease
Dex: dexmedetomidine
I/R: ischemia/reperfusion
HE: hematoxylin-eosin
TUNEL: terminal deoxynucleotidyl transferase dUTP nick-end labeling
IHC: immunohistochemistry
RM: rotary microtome
CD34: CD34 molecule
vWF: von Willebrand factor
MEM: minimum essential medium
FBS: fetal bovine serum
CCK-8: cell counting kit-8
FITC: fluorescein isothiocyanate
Bcl-2: BCL2 apoptosis regulator
Bax: BCL2-associated X
LC3: light chain 3
Beclin-1: beclin 1
